# Extensive innate immune gene activation accompanies brain aging, increasing vulnerability to cognitive decline and neurodegeneration: a microarray study

**DOI:** 10.1186/1742-2094-9-179

**Published:** 2012-07-23

**Authors:** David H Cribbs, Nicole C Berchtold, Victoria Perreau, Paul D Coleman, Joseph Rogers, Andrea J Tenner, Carl W Cotman

**Affiliations:** 1 Institute for Memory Impairments and Neurological Disorders, University of California, Irvine, 1226 Gillespie NRF, Irvine, CA, 92697, USA; 2 Department of Neurology, University of California, Irvine, 1226 Gillespie NRF, Irvine, CA, 92697, USA; 3 Centre for Neuroscience, University of Melbourne, Parkville, VIC, 3010, Australia; 4 Center on Aging and Developmental Biology, University of Rochester Medical Center, 601 Elmwood Ave, Rochester, NY, 14642, USA; 5 Sun Health Research Institute, L. J. Roberts Center for Alzheimer's Research, 10515 West Santa Fe Drive, Sun City, AZ, 85372, USA; 6 Departments of Molecular Biology and Biochemistry, University of California, Irvine, 1226 Gillespie NRF, Irvine, CA, 92697, USA; 7 Deparment of Neurobiology and Behavior, University of California, Irvine, 1226 Gillespie NRF, Irvine, CA, 92697, USA; 8 Institute for Immunology, University of California, Irvine, 1226 Gillespie NRF, Irvine, CA, 92697, USA

**Keywords:** Complement, Toll-like receptor, Inflammasome, Cryopyrin, Caspase-1, Myeloid-related protein, Calgranulin, Calprotectin, Alarmin, Endogenous danger signaling, Fractalkine

## Abstract

**Background:**

This study undertakes a systematic and comprehensive analysis of brain gene expression profiles of immune/inflammation-related genes in aging and Alzheimer’s disease (AD).

**Methods:**

In a well-powered microarray study of young (20 to 59 years), aged (60 to 99 years), and AD (74 to 95 years) cases, gene responses were assessed in the hippocampus, entorhinal cortex, superior frontal gyrus, and post-central gyrus.

**Results:**

Several novel concepts emerge. First, immune/inflammation-related genes showed major changes in gene expression over the course of cognitively normal aging, with the extent of gene response far greater in aging than in AD. Of the 759 immune-related probesets interrogated on the microarray, approximately 40% were significantly altered in the SFG, PCG and HC with increasing age, with the majority upregulated (64 to 86%). In contrast, far fewer immune/inflammation genes were significantly changed in the transition to AD (approximately 6% of immune-related probesets), with gene responses primarily restricted to the SFG and HC. Second, relatively few significant changes in immune/inflammation genes were detected in the EC either in aging or AD, although many genes in the EC showed similar trends in responses as in the other brain regions. Third, immune/inflammation genes undergo gender-specific patterns of response in aging and AD, with the most pronounced differences emerging in aging. Finally, there was widespread upregulation of genes reflecting activation of microglia and perivascular macrophages in the aging brain, coupled with a downregulation of select factors (TOLLIP, fractalkine) that when present curtail microglial/macrophage activation. Notably, essentially all pathways of the innate immune system were upregulated in aging, including numerous complement components, genes involved in toll-like receptor signaling and inflammasome signaling, as well as genes coding for immunoglobulin (Fc) receptors and human leukocyte antigens I and II.

**Conclusions:**

Unexpectedly, the extent of innate immune gene upregulation in AD was modest relative to the robust response apparent in the aged brain, consistent with the emerging idea of a critical involvement of inflammation in the earliest stages, perhaps even in the preclinical stage, of AD. Ultimately, our data suggest that an important strategy to maintain cognitive health and resilience involves reducing chronic innate immune activation that should be initiated in late midlife.

## Background

The role of inflammation in brain health is a major focal point of contemporary research in aging and Alzheimer’s disease (AD) [1-4], and activation of inflammatory pathways in the brain is increasingly emphasized as a major risk factor for the development and progression of AD [2,5,6]. Consistent with the idea that activation of the immune system in the brain is harmful, the majority of recent studies in transgenic mouse models of AD support the notion that immune activation can precipitate the onset of AD-like pathology [5-10] (but see [11-13]). In addition, epidemiological studies in humans suggest that long-term use of anti-inflammatory drugs during adult life protects brain function and delays the onset of cognitive decline [5,14,15], with animal studies providing additional support for this hypothesis [16-18]. In contrast, clinical studies attempting to treat AD with anti-inflammatory drugs once the disease is clinically apparent have been largely unsuccessful [19-22]. Taken together, these studies suggest that the timing of anti-inflammatory treatment is crucial, and that attenuation of inflammation is particularly important prior to clinical manifestation of AD.

While immune activation in the brain is an accepted part of AD pathogenesis, it has generally been assumed that such neuroinflammation is minimal in cognitively normal aging. However, the findings that amyloid-beta (Aβ) deposits are associated with inflammatory proteins and microglia in the early stages of AD pathology [2,23,24]), coupled with the recent data that volume density of microglia is increased already in cognitively normal subjects who have frequent presence of plagues and tangles [24,25], has led to the hypothesis that there may be critical involvement of inflammation already in the preclinical stages of AD [26,27]. Further, our recent data suggest that neuroinflammation is present in the brain well prior to cognitive decline. Using microarray analysis to identify functional classes of genes showing altered expression in cognitively normal aging across the adult lifespan (age 20 to 99 years), we found that immune activation is a highly prominent feature of cognitively normal brain aging [28]. If immune and inflammation-related genes are activated in the brain in the course of cognitively normal aging, it is possible that such neuroinflammation primes the brain for neurodegenerative cascades, cognitive decline, and progression to AD [29-31].

The extent to which immune/inflammation-related genes are activated in the brain in normal aging or AD has not been examined in a comprehensive manner. Thus, this study undertakes a systematic and comprehensive analysis of gene expression profiles of this class of genes in a well-powered microarray study of young, aged, and AD cases to address a number of questions. In particular, do immune and inflammation-related genes undergo a progressive preclinical activation in aging or undergo a precipitous upregulation in AD? What are the response profiles of genes involved in the innate immune response, such as the complement pathway, toll-like receptor (TLR) signaling, inflammasome activation, and other markers indicative of microglial activation, and how do these response profiles compare in aging and AD? Are there gender differences in the responses of immune/inflammation genes in aging or AD? Finally, because brain regions are differentially vulnerable to accumulating pathology in aging and AD, we examined gene expression profiles of immune/inflammation genes in four brain regions. Three of these regions, the hippocampus (HC), entorhinal cortex (EC), superior frontal gyrus (SFG), are critical for higher cognitive function and develop AD-type neuropathology, with associated functional declines, in both aging and AD. In contrast, the fourth brain region, the post-central gyrus (PCG) is a somatosensory cortical region that appears to be relatively refractory to developing pathology. Our study is the first to use the microarray approach to evaluate gene expression changes in immune/inflammation-related genes in relationship to age, gender, brain region, and the development of AD, and provides a detailed focus on key sets of inflammatory genes associated with the innate immune response.

## Materials and methods

### Case selection, group classification, and brain region designation

Frozen unfixed tissue was obtained from 57 neurologically and cognitively normal controls (age 20 to 99 years) and from 26 Alzheimer’s disease cases (age 74 to 95 years) using tissue banked at seven well-established National Institute on Aging Alzheimer’s Disease Center (ADC) brain banks located at the University of California, Irvine, Sun Health Research Institute, University of Rochester, Johns Hopkins University, University of Maryland, University of Pennsylvania, and the University of Southern California. Tissue was obtained from four brain regions: the entorhinal cortex (EC), hippocampus (HC), superior frontal gyrus (SFG), and post-central gyrus (PCG), using the landmarks described previously to standardize dissection of the four brain regions [28]. Tissue was available from two or more regions from 85% of the cases, resulting in a total of 240 tissue samples (50 EC, 64 HC, 64 PCG, 62 SFG). Group sizes were as follows: young (n = 22, 20 to 59 years, mean age 35.4 ± 10.5 years), aged controls (n = 33, age 69 to 99, mean age 84.2 ± 8.9 years), and AD cases (n = 26, ages 74 to 95 years, mean age 85.7 ± 6.5 years) with males and females similarly represented in each group. Individual case details are shown in Additional file 1: Table S1.

### Clinical and neuropathological criteria

Clinical and neuropathological data were available for all cases aged 60 to 99. Controls had no memory complaints or history of memory complaints, with normal cognitive function documented by scoring within 1.5 standard deviations of the age and education adjusted norms. Mini-mental status examination (MMSE) scores for controls ranged from 25 to 30 (average = 28.35 ± 1.57), and global clinical dementia rating (CDR) = 0 for all cases. AD cases were characterized by a progressive decline in memory, cognitive deficits in two or more areas, MMSE < 24 (average = 18.32 ± 9.19), CDR = 2 to 3, Braak stage II to VI (average stage IV), and neuropathological diagnosis of AD based on CERAD and NIA/Reagan Institute criteria.

Clinical exclusion for both controls and AD included evidence of Alcoholism (DSM-IV or pattern of pathological use), co-existing major psychiatric illness or major depression (Hamilton depression rating), brain damage from an earlier known cause (for example, hypoxia, coma, head trauma), cancer with evidence of metastasis to the brain or wide disease metastasis, or history of cerebral vascular disease. Neuropathological exclusion criteria for both controls and AD included the following: (1) dementia with Lewy bodies, Parkinson’s disease or other non-AD dementias, (2) hippocampal sclerosis, (3) gross infarcts in excess of 50 ml total or smaller infarcts in areas of interest, (4) recent or old intracerebral hemorrhage in excess of 25 ml or subarachnoid hemorrhage exceeding 10 ml, (5) sepsis, meningitis, encephalitis, or pathologic evidence of metabolic brain disease, anoxia, drug intoxication or alcoholism, (6) primary or metastatic tumor except for small, incident meningioma, (7) extensive white matter lesions, (7) Binswanger's disease or multiple sclerosis. Data enabling exclusion criteria for subject previously taking over-the-counter or prescription nonsteroidal or steroidal anti-inflammatory drugs were not available.

### Tissue processing, gene-chip hybridization, and quality control

Total RNA was extracted from the frozen, unfixed tissue using Trizol reagent (Invitrogen, Carlsbad, CA, USA), and purified using quick spin columns (Qiagen, Valencia, CA, USA). RNA quality was assessed using the Agilent Bioanalyzer (Agilent Technologies, Palo Alto, CA, USA), with average RIN = 8.3 ± 0.7 across all samples (Additional file 1: Table S1). Each sample was individually hybridized to high-density oligonucleotide gene chips from Affymetrix (Human genome Hg-U133 plus 2.0). These chips measure the expression of > 50,000 transcripts and variants, including 38,500 characterized human genes. Gene chips were processed at the Core Facility at UC Irvine using a robotic system and following manufacturer’s recommendations. Briefly, total RNA (10 ug) from each sample was used to generate first strand cDNA using a T7-linked-(dT)_24_ primer, followed by *in vitro* transcription using the ENZO BioArray HighYield RNA transcript labeling kit (ENZO, Farmingdale, NY, USA) to generate biotin-labeled cRNA target. Using a robotics system (Biomek FX MicroArray Plex SA System; Beckman Coulter, Brea, CA. USA) to optimize consistency in processing and minimize handling variability, each fragmented, biotin-labeled cRNA sample (30 ug) was individually hybridized to an Affymetrix Hg-U133 plus 2.0 chip for 16 hours and rotated at 13 rpm at 50°C. The chips were washed and stained on a fluidics station and scanned. After scanning, CEL files were assessed manually for grid alignment and to ascertain absence of scratches and bubbles. Quality control on the chips was assessed using Affymetrix Quality Reporter software. Background signal, average signal present, percentage of probe sets called Present, spike-in controls BioB and BioC, and housekeeping genes GAPDH (3/5 ratio), HS-HUMISGF3A (3/5 ratio), and HS-HSAC07 (3/5 ratio) were assessed, and only arrays where all quality control values were within acceptable range (mean ± 1 standard deviation) were included for further analysis.

### Microarray analysis

Using GeneSpring 7.3.1 software (Agilent Technologies, Palo Alto, CA, USA), expression values for each probe set were calculated from CEL files using GC-RMA, an algorithm based on the Robust Multiarray Average (RMA) software by Irizarry *et al.*. [32,33]. GC-RMA takes into consideration the binding efficiency of the probes based on the guanine and cytosine (GC) contents of the probes, and incorporates the MisMatch (MM) feature of Affymetrix microarrays, which is intended to measure nonspecific binding. This model-based algorithm incorporates information from multiple microarrays to calculate the expression of a gene. The probe response pattern is fitted over multiple arrays using an additive model. The fitted model detects abnormally behaving probes, which are subsequently excluded for calculating gene expression. After extracting probe set raw signal intensity values from gene chip CEL file, default settings were applied for per-chip and per-gene normalization, and expression values underwent log-transformation of the geometric mean followed by statistical analysis for significant probe sets.

### Statistical analysis (GeneSpring)

An initial list of immune- and inflammation-related genes was generated based on the Gene Ontology categories ‘defense response (GO:6952)’ (which contains GO subcategories of inflammatory response, innate immune response, positive and negative regulation of the defense response, and defense response to pathogens), supplemented by manual curation. 1532 probe sets on the Affymetrix Hg-U133 plus 2.0 microarray represent this functional category, which are referred to as immune/inflammation-related genes in this paper. To limit the analysis to mRNAs that were present at levels that were reliably detectable by the microarray, the immune/inflammation-related probe sets were then filtered on Flag detection calls to remove probe sets that were absent on more than 50% of the chips for a given region, as described previously [28]. This threshold removes probe sets with unreliable signal and very effectively reduces the incidence of false positives [34], and in this case reduced the target list size from 1532 probesets to 759 reliably detectable probe sets related to immune/inflammation function. These 759 probe sets were then analyzed for expression changes in aging or AD, with significance threshold set at *P* < 0.01 and variance measures based on the cross-gene error model in GeneSpring as described previously [28]. The probe sets and genes interrogated are listed in Additional file 1: Table S1. Aging-AD continuum genes were identified as genes for which there was a strong linear relationship between young, aged, and AD groups, and for which the direction of change remained consistent across young versus aged and aged versus AD. A two-step procedure was used, first identifying immune/inflammation genes for which the regression across all three comparison groups (young, aged, AD) was significant with a threshold of *P* < 0.01. Second, using this subset we identified only those genes that demonstrated a consistent pairwise relationship as defined by the upper and lower bounds of the corresponding confidence interval for the difference in means for each pairwise comparison, using 90% confidence limits. The 90% threshold following linear regression based on *P* < 0.01 was selected to balance the probability of either type I or type II errors. Identification of continuum genes was first based on analysis with gender pooled, followed by analysis of gender-specific changes in each region.

### Select gene lists

Many of the gene changes that emerged in the analysis of immune/inflammation genes were associated with the innate immune response. Based on this, we looked in detail at changes in probe sets related to major complement pathway elements, toll-like receptors (TLR) and related molecules, complement pathway elements, caspase-1 and inflammasome-related genes, modulators of microglial and perivascular macrophage activation, chemokine ligands and receptors, scavenger and immunoglobulin receptors, and major histocompatibility classes of genes (MHCI, MCH II). Gene lists were established by searching the immune/inflammation gene list by keyword for the relevant functional class, resulting in lists containing the following: 22 probe sets representing 16 complement-related genes, 20 probe sets representing 16 TLR-signaling related genes, 13 probe sets representing 6 genes related to inflammasomes, 14 probe sets representing 10 chemokine ligands and receptors, 12 probe sets representing 9 scavenger and immunoglobulin receptors, 20 probesets representing 8 major histocompatibility class (MHC) I genes, and 19 probesets representing 13 MHC II genes. Only probesets that had a present call >50% across all microarrays for a given region were included. Significant changes were identified using a statistical threshold of *P* < 0.01. For a complete list of immune-related probesets interrogated, with corresponding fold changes, please contact the authors.

### QPCR validation of age- and AD-associated changes in a subset of genes identified by gene chip analysis

A subset of inflammation-related genes was further analyzed by qPCR to validate the age- and AD-related changes derived from the gene chip studies. The genes assessed in hippocampal tissue by qPCR were CD14, TLR2, TLR4, TLR7, TOLLIP (an inhibitor of TLR signaling), MYD88 (a positive modulator of TLR signaling), and the cytokines TNF alpha, interleukin-1 beta (IL-1β), IL-6, and IL-10 and a GUSB (beta glucuronidase) endogenous control assay. The sample sizes for PCR analysis of HC gene expression were based on availability of RNA after running the microarrays (young, n = 10; aged, n = 11; AD, n = 4). Inflammasome-related gene expression was quantified in SFG tissue based on availability of RNA after running the microarrays (young, n = 4; aged, n = 8; AD, n = 11) for NRLP3 and ASC, using commercially available probes (SABiosciences, Frederick, MD, USA). Appropriate control genes for the SFG were determined using NormFinder, with the following combinations of control genes identified as optimal: cofilin 2 and FBOX42 for the aging analysis, and cofilin 2 and SLC25a16 for the comparison of AD to age-matched controls. Quantitative PCR was performed using TaqMan Gene Expression Assays (Applied Biosystems, Foster City, CA, USA). Each TaqMan Gene Expression Assay is preformulated consisting of an unlabeled forward and reverse primer at a final concentration of 900 nM and 1 FAM™ dye-labeled TaqMan™ MGB of 250 nM final concentration. Sequences of the primers and probe are proprietary information. For each sample, 3.0 ug total RNA was reverse transcribed using the High Capacity cDNA Reverse Transcription Kit (Applied Biosystems). Two ml of a 1:5 dilution for cDNA was combined with TaqMan Universal PCR Master Mix No AmpErase UNG (Applied Biosystems) and the TaqMan Gene Expression Assay in a 10 ml reaction set up by the CAS-1200 liquid handling system. The real-time qPCR amplifications were run on an ABI PRISM 7900 HT Sequence Detection System (Applied Biosystems). Universal thermal cycling conditions were as follows: 10 minutes at 95 degrees C, 40 cycles of denaturation at 95 degrees C for 15 seconds, and annealing and extension at 60 degrees C for 1 minute. Amplifications efficiencies were close to 100% for all assays according to analyses of a number of different dilutions of the cDNA. Calculations were done assuming that 1 delta Ct equals a two-fold difference in expression.

## Results

Previously, our microarray data from a genome-wide microarray study investigating gene expression changes in the brain across the adult lifespan revealed that immune activation is a prominent feature of cognitively normal aging [28]. The present study builds on this initial observation to analyze the extent to which gene expression of 759 immune/inflammation-related probe sets is altered in the course of aging and AD, in four brain regions (the HC, EC, SFG and PCG). Of these brain regions, the HC, EC and SFG are vulnerable to neuropathology accumulation and functional decline with age and AD, while the PCG is relatively spared. Subsequently, we focus on gene responses of key sets of inflammatory markers associated primarily with the innate immune response.

### More extensive response in aging than AD, with a subset of genes undergoing progressive change across aging and AD

**Figure 1:**
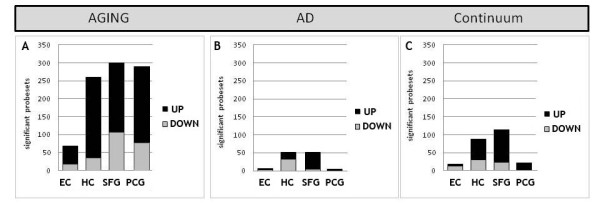
**Immune-related genes undergo more extensive response in the course of cognitively normal aging (age 20 to 99) than in Alzheimer’s disease (AD).** (**A**) In aging, comparing gene expression levels in young (20 to 59 yrs) versus aged (60 to 99 yrs) individuals revealed that numerous immune-related gene changes occur in the superior frontal gyrus (SFG), post-central gyrus (PCG) and hippocampus (HC), with fewer genes showing significant change in the entorhinal cortex (EC) with age. The majority of gene responses were increased expression with age, in all brain regions assessed. (**B**) Relative to aging, fewer immune genes showed significant change in AD versus age-matched controls, with gene responses primarily restricted to the HC and SFG. Negligible immune gene expression change was observed in the EC and PCG in AD. (**C**) A subset of immune-related genes underwent progressive change across aging and AD, particularly in the HC and SFG, predominantly undergoing increased expression across aging and AD. Few immune genes underwent progressive change across aging and AD in the EC and PCG.

To identify the extent to which immune/inflammation-related genes are affected over the course of aging, expression levels of the 759 probe sets were compared in young (age 20 to 59) versus aged controls (60 to 99) in each of the four target brain regions. This analysis revealed an unexpectedly large number of significant transcriptional changes in immune/inflammation-related genes, with predominant gene upregulation and a region-specific response profile (Figure 1A). The greatest number of genes responded in the HC and the cortical regions (SFG and PCG), with approximately 40% of interrogated probe sets showing altered expression, while few immune/inflammation-related genes showed significant age-related change in the EC. In all brain regions, the majority of responding immune/inflammation-related genes was upregulated with age (64 to 84%, depending on brain region) (Figure 1A).

Changes in immune/inflammation gene expression were next assessed in AD cases relative to age-matched controls (*P* < 0.01), with the prediction that extensive change would be apparent in the HC, EC, and SFG, and relatively little in the PCG. Further, we predicted that more immune/inflammation genes would undergo transcriptional change in AD than in aging. Contrary to our predictions, AD was associated with far less extensive response of immune/inflammation genes than was observed over the course of aging. Of the 759 immune/inflammation-related probe sets interrogated, only 6% were significantly altered in AD relative to age-matched controls (Figure 1B). In addition, gene responses were primarily detected in the HC and SFG in AD, with virtually no immune/inflammation genes identified as significantly changed in the EC in AD, and similarly few genes responding in the PCG. Nearly all the significant immune/inflammation genes in the SFG were upregulated in AD, while in the HC, a greater proportion of significant genes was downregulated (Figure 1B).

A key unknown in the fields of aging and AD is whether transcriptional changes in immune/inflammation genes apparent in the AD brain are initiated to some degree already in aging. To address this possibility, we evaluated the extent to which immune/inflammation-related genes in a given region undergo progressive upregulation or downregulation across aging and AD. Linear regression analysis (*P* < 0.01) was applied across the three groups (young, aged, AD), followed by elimination of genes that did not show a consistent directional change across aging and AD, based on a 90% confidence interval threshold. Genes meeting these criteria were designated ‘continuum’ genes. This analysis revealed that only a subset of immune/inflammation genes met the criteria for progressive change, with the greatest number in the SFG and HC, and relatively few genes showing this pattern of expression change in the EC or PCG (Figure 1C). The majority of significant ‘continuum’ genes in the SFG and HC showed progressive upregulation (79% and 66% respectively), with relatively fewer progressively downregulated over aging and AD (21% and 34% respectively).

Overall, these analyses reveal that immune/inflammation genes undergo more extensive responses in the course of normal aging than in AD. Some immune/inflammation-related genes undergo progressive change across aging and AD, especially in the HC and SFG (AD-vulnerable regions), suggesting that a subset of the genes that change in AD have already been initiated to some degree in normal aging. Overall, the EC showed very limited expression change of immune/inflammation genes in either aging or AD.

### Region-specific patterns of change in males and females

While the importance of gender in modulating gene responses has become increasingly recognized in the last few years, gender-specific patterns of gene change that may occur in the brain in aging and AD have only scarcely been addressed. Our previous analysis suggested that gender affected the extent of immune/inflammation gene change that occurred in the brain over the course of aging. Building on this initial observation, here we analyze in detail gender-specific patterns of immune/inflammation gene response in various brain regions in aging and AD.

**Figure 2:**
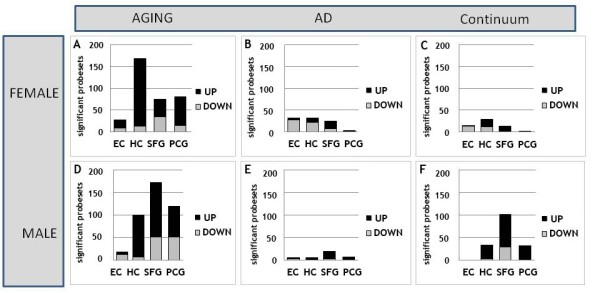
**Immune-related genes undergo gender and region-specific patterns of response in aging and Alzheimer’s disease (AD).** In both females and males, the extent of immune-gene response is greater in aging (**A**, **D**) than in AD (**B**, **E**), with gender-specific patterns of response across brain regions. In aging, females show the greatest number of genes responding in the hippocampus (HC) (A), while the most responsive region in males was the superior frontal gyrus (SFG) (D). In both genders, the entorhinal cortex (EC) showed the fewest numbers of responding genes over the course of aging, the post-central gyrus (PCG) underwent an intermediate response, and the direction of gene change was predominantly upregulated in all brain regions. In AD (**B**, **E**), males and females both showed a limited number of significant gene responses, with a greater number of significant gene changes observed in females (B) relative to males (E), particularly in the EC and HC. For genes undergoing progressive change across aging and AD (**C**, **F**), few such genes were apparent in females (C), with a relatively large number genes following this pattern in the SFG in males, with the majority of these genes undergoing progressively increased expression across age and AD

In both males and females, in all four brain regions, the extent of immune/inflammation gene change was greater in aging than in AD. However, the brain regions showed gender-specific patterns of change, particularly in aging. In females, the most extensive age-related gene change was apparent in the HC, while in males the most responsive region was the SFG (Figure 2A). In both males and females, the EC showed the least numbers of responding genes over the course of aging while the PCG underwent an intermediate extent of gene change, and the direction of gene change was predominantly upregulated in aging.

In AD, while males and females both showed a limited number of significant gene responses, several gender-specific differences in gene expression patterns were apparent. Notably, a greater number of significant gene changes were observed in females relative to males in AD, especially in the EC and HC. In addition, the direction of gene change in all brain regions was virtually exclusively upregulated in males, while in females a majority of genes were downregulated, notably in the limbic regions (Figure 2B).

Finally, gender-specific patterns in immune/inflammation gene responses also emerged when analyzing genes undergoing progressive change across aging and AD in males and females. Notably, while few genes showed this pattern of response in any brain region in females, a relatively large number of ‘continuum’ genes were detected in the SFG in males, with the majority of these genes undergoing progressively increased expression across age and AD (Figure 2C).

Overall, these data reveal that the class of genes related to immune/inflammation function show gender-specific patterns of change in aging and AD, with the most pronounced differences emerging in aging. Analysis of the region-specific patterns of response in aging and AD indicate that the HC is relatively more prone to immune/inflammation gene responses in females than in males, while the SFG is more susceptible to immune/inflammation gene responses in males.

### Analysis of aging- and AD-related changes in specific gene classes associated with inflammation and innate immunity

Functional analysis of the immune genes responding in age and AD revealed that many of the immune/inflammation-related genes were associated with the innate immune response. The innate immune response represents a first line of defense to pathogens and other stimuli. The main effector classes of the innate immune response are the complement pathway, TLR signaling, inflammasome activation, and scavenger and immunoglobulin receptors. These different families act both redundantly and in concert to organize a generic response to non-self pathogens, and to stimulate adaptive immune responses involving MHC I and MHC II molecules [35,36]. Microglial and perivascular macrophages are the principal effector cells driving the innate immune response in the brain. There is increasing evidence that many aspects of the innate immune response can be activated not only by pathogens but also by endogenous factors that accumulate with age and AD. For example, amyloid-beta (Aβ) has been shown to activate the complement cascade via both the classical and alternate pathways [37,38], to activate toll-like signaling (via activation of TLR2 and TLR4), and to activate the inflammasome, all of which lead to release of proinflammatory factors [37,39-49]. Innate immune activation can be neuroprotective when it is activated appropriately in response to non-self pathogens and in an acute fashion [4,50-53]. However, chronic activation of the innate immune response in the brain is thought to contribute to neurodegeneration in AD [30,54]. Little is known about the changes in the innate immune response in humans in the course of cognitively normal aging.

To investigate if the innate immune system is engaged in the brain in normal aging, and to compare the extent of engagement in aging and AD, we analyzed in greater detail expression patterns of genes related to the complement pathway, TLR signaling, inflammasome activation, scavenger and immunoglobulin receptors, and MHC I and II.

### Aging- and AD-associated increased gene expression of complement pathway proteins

The complement system is a critical component of the innate immune response. The involvement of complement activation in the pathogenesis of AD has been extensively investigated since the original observations by Eikelenboom and colleagues [55,56]. In particular, activation of complement and the associated inflammatory signaling, opsonization, and cellular damage due to the membrane attack complex have been proposed to contribute to AD pathogenesis [37,56-58].

Analysis of complement-related genes revealed primarily upregulation of these genes with aging. Among the genes showing an increase in expression were several complement receptors and numerous components of the classical and alternative complement pathways (Additional file 2: Table S2A). Complement-related gene upregulation was pronounced in the HC, SFG and PCG, with more modest response in the EC. In particular, aging was associated with upregulation of C1qA, C1qB, C1qC, C1s, C3, C3a receptor 1 (C3AR1), C4α, C4β, C5, C5a receptor 1 (C5AR1), gene expression in the HC, SFG and PCG. In parallel, genes that curtail complement activation (factor H (CFH), CFH-related 1 (CFHR1), and clusterin) were also upregulated in the HC, SFG and PCG with aging. In the EC, complement genes upregulated with age included C1qB, C1qC, C4a and clusterin.

In AD, only a subset of complement-related genes showed altered expression relative to age-matched controls (Additional file 2: Table S2B). Specifically, C4A and C4B were significantly upregulated in AD in the HC and EC and C3AR1 and C5AR1 in the SFG, while no complement-related genes were significantly different in the PCG region. These complement genes also showed a significant progressive upregulation across aging and AD (‘continuum’ genes), along with CFHR1 and clusterin in the HC (Additional file 2: Table S2B). Interestingly, the continuum analysis revealed significant progressive upregulation of C5AR1 in the PCG as well. Only one gene that was significantly changed in AD did not show a parallel response in aging: in the HC, C1q binding protein (C1QBP) was significantly downregulated in AD, but was upregulated in aging.

Overall, these analyses reveal that complement genes are broadly upregulated in aging, undergo more extensive responses in the course of normal aging than in AD, and that only a small subset of complement-related genes show progressive change across aging and AD. Increased expression of complement genes in aging is potentially a response to an increase in extracellular debris, such as Aβ [59]. Importantly, elevated complement expression may facilitate clearance of such extracellular debris, the effects of which may be protective. However, chronic complement activation is likely to be harmful due to release of potent inflammatory peptides (such as anaphylatoxins C3a and C5), that bind their activating receptors C3aR1 and C5aR1 (CD88), and formation of the membrane attack complex, which can damage cell membranes [60].

### Aging- and AD-associated changes in gene expression of TLRs and associated proteins

The TLR system is another key effector mechanism of the innate immune defense system. TLRs initiate signaling cascades that lead to the production of a wide array of proinflammatory mediators including cytokines, chemokines, and reactive oxygen/nitrogen species. This system of innate immunity is based on a series of pattern recognition receptors, which recognize pathogen-associated molecular patterns [61]. In addition to activation by non-self pathogenic molecules, TLR signaling can be activated by endogenous ligands including molecules that accumulate with age and AD, such as Aβ, and other ligands that are released by injured cells [62-66].

Analysis of TLR-related gene expression revealed a robust upregulation of this gene class in aging, particularly in the HC, PCG and SFG, with less extensive response in the EC (Additional file 3: Table S3A). TLR2 was upregulated in all four regions with age, with upregulation of TLR4, TLR5, and MYD88 apparent in the HC, SFG, and PCG. Additional age-related upregulation of TLRs was apparent in the HC (TLR1, TLR3, TLR 7, TLR8). In AD, receptors associated with TLRs were not as extensively changed as in aging, similar to the pattern observed for the complement system. Several TLR-related genes show progressive continuum of change across aging and AD, including upregulation of TLR4 and TLR5 in the HC and SFG, and TLR2 in the SFG (Additional file 3: Table S3B). Only one gene that was significantly changed in AD did not show a parallel response in aging: TLR 7 is significantly upregulated in the SFG in AD, but shows little change in aging. Overall, genes that promote TLR signaling are broadly upregulated in the brain, most prominently in the course of aging, with a subset of TLR genes showing additional change in the AD brain.

Recent findings demonstrate that TLR signaling can be activated by endogenous nonpathogenic ligands, in particular endogenous molecules released in response to injury or inflammation [67]. One such ligand is calprotectin, which consist of a heterodimer of two members of the S100 calcium-binding family of proteins, S100A8 and S100A9, also respectively know as myeloid-related protein 8 (MRP8) and 14 (MRP14), that can act in synergy with endogenous and exogenous danger signals to promote inflammation via TLR interaction [65,68,69]. Analysis of S100A8 and S100A9 gene expression revealed that aging was associated with marked upregulation (four- to thirteen-fold) of S100A8 in all four brain regions and upregulation of S100A9 (two- to three-fold) in the SFG and PCG. Similarly, CD14, a co-activator of TLR2 and TLR4, was upregulated in all four regions with aging. While aging was accompanied by widespread upregulation of calprotectin and CD14, only the S100A8 component of calprotectin showed further upregulation in AD (SFG) and CD14 showing no significant change in any region in AD.

In parallel with increased expression of TLRs, CD14, and endogenous activators of TLR signaling, expression of TOLLIP (a toll-interacting protein that attenuates TLR signaling) was downregulated in multiple brain regions in both aging and AD. TOLLIP was significantly downregulated in the SFG and PCG in aging (in Additional file 3: Table S3B) and underwent a significant progressive downregulation across aging and AD in the EC, HC and SFG. Such downregulation of TOLLIP suggests that the ‘brakes’ on TLR signaling are less accessible with age and AD.

Taken together, these analyses reveal that genes that promote TLR signaling are broadly upregulated in aging, and undergo more extensive responses in the course of normal aging than in AD, similar to the response seen for complement-related genes. A subset of TLR genes undergoes progressive change across aging and AD, particularly in the HC and SFG, regions vulnerable to decline in AD. Similar to the consequences of complement activation, TLR signaling activates downstream inflammatory processes, and chronic upregulation of the TLR system potentially promotes a harmful proinflammatory environment in the brain. While there is evidence that TLR signaling can mediate some beneficial effects in CNS [54,70], it is believed that TLR-induced activation of microglia and release of proinflammatory molecules are responsible for neurotoxic processes in the course of various CNS diseases including AD [71].

### Divergent expression of caspase-1 and inflammasome-related genes in aging and AD

Another component of the innate immune response involves activation of molecular platforms known as ‘inflammasomes’. Inflammasomes are multi-molecular complexes that bind to procaspase-1 to activate the caspase-1 cascade, leading to the maturation of the proinflammatory cytokines interleukin 1β (IL-1β) and IL-18 [72-74]. IL-1β and IL-18 are elevated in the AD brain [2,75-77] and have been hypothesized to contribute to neurodegeneration and cognitive decline in AD [76,78]. Production of these proinflammatory cytokines is highly regulated. While availability of the inactive precursor molecules (pro-IL-1β and pro-IL-18) is increased in response to TLR signaling and cytokines, processing by caspase-1 is required to form the active cytokines, thus positioning the inflammasome as a key regulatory step controlling release of these potent proinflammatory agents. To investigate if inflammasome-related genes show altered expression in aging or AD, we investigated expression patterns for caspase-1 and its downstream targets IL-1β and IL-18, expression of key components of the inflammasome complex (NLRP3, ASC), and expression of several genes involved in inflammasome activation, including thioredoxin-interacting protein (TXNIP), P2X7, and pannexins.

**Figure 3:**
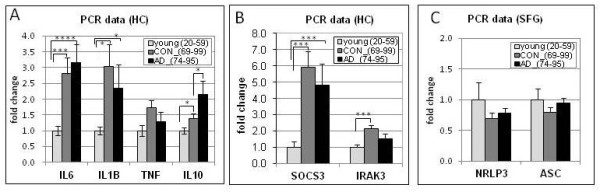
**qPCR was used to assess expression profiles of several genes for which gene expression was below microarray detection sensitivity.** qPCR analysis of hippocampal gene expression for the proinflammatory cytokines IL-6, IL-1beta, NF-alpha, and the anti-inflammatory cytokine IL-10 revealed significant upregulation of IL-6, IL-1beta and IL-10 with age, and further upregulation of IL-10 in Alzheimer’s disease (AD) (**A**). Similarly, modulators of cytokine signaling were upregulated in the hippocampus (HC) with age including a six-fold increase in the suppressor of cytokine signaling (SOCS-3) and a two-fold increase in IRAK3, a serine/threonine kinase that mediates signaling from toll-like receptors (TLRs) and IL-1 receptor family members (**B**). In contrast, no significant change in components of the inflammasome (NRLP3 and ASC) was detected in the superior frontal gyrus (SFG) with aging or AD (**C**). **** *P* < 0.0001, ***P < 0.005, *P < 0.05.

Assessment of inflammasome-related genes revealed prominent upregulation of several inflammasome-related genes, particularly in aging (Additional file 4: Table S4A, B). Caspase-1 upregulation was apparent in the HC, PCG and SFG in aging, with gene expression in the SFG undergoing progressive upregulation across aging and AD. In addition, IL-18 was upregulated in aging in the HC and PCG. While IL-1β expression was below detection by the microarray, qPCR analysis of HC tissue revealed upregulation of IL-1β with aging in the HC, with no further upregulation in AD (Figure 3A). Interestingly, while caspase-1 (the effector of inflammasome action) was prominently upregulated with aging, other components of the inflammasome complex did not show parallel changes with aging or AD. qPCR analysis of NRLP3 and ASC in SFG tissue revealed that gene expression was not significantly changed for these inflammasome components, either in aging or AD, although there appeared to be a decline in aging (Figure 3C). Finally, recent literature has revealed that inflammasomes can be activated via different factors, with key roles for TXNIP, pannexins, and P2X7. Expression of TXNIP and pannexins 1 and 2 was prominently upregulated in multiple brain regions in aging, notably the EC, PCG, and SFG, with pannexins 1 and 2 showing progressive upregulation across aging and AD in the HC.

Taken together, aging was characterized by widespread increased expression of caspase-1, upregulation of targets of caspase-1 action (Il-1β, IL-18), and increased expression of agents that can activate the inflammasome, suggesting that the inflammasome is poised for increased activity with age. Like the TLR system, inflammasomes can be activated by endogenous factors such as Aβ, as well as by oxidative stress [49,72,79], brain levels of which increase with advanced age. Thus, inflammasome activation can trigger a cascade of events that have the potential to activate multiple arms of the innate immune system and drive a chronic proinflammatory state in the elderly brain.

### Modulators of microglial and perivascular macrophage activation in aging and AD

Microglial and perivascular macrophages are the principal effector cells driving the innate immune response in the brain. While microglia and macrophages can serve a neuroprotective function when they participate in the elimination of cellular debris, chronic activation is thought to be a major contributor to cognitive decline and neuropathogenesis in various neurodegenerative disorders including AD [80,81]. Microglia express receptors for immunoglobulins (FcRs), which trigger effector functions such as production of cytokines and chemokines, phagocytosis and degranulation. FcRs are upregulated in response to innate immune activation, and are increasingly recognized for their contributions to the pathogenesis of neurological disorders [82-84].

To investigate how these aspects of microglial and macrophage activation are affected in aging and AD, we analyzed the gene expression profiles of FcRs and the CXC and CCL classes of chemokine ligands and receptors. Analysis of FcR gene expression revealed widespread upregulation of both low and high affinity FcRs, particularly in aging. Notably, aging was characterized by upregulation of CD64, CD32, CD16b, and FCϵR1a in the HC, PCG and SFG, with a subset of these immunoglobulin receptors also increased in the EC with aging (Additional file 5: Table S5A). In addition, CD64 and CD16b showed progressively increased expression in the SFG across aging and AD (Additional file 5: Table S5B). In contrast to the robust response profiles of the FcRs, little change was detected in the CXC and CCL classes of chemokine ligands and receptors, in either aging or AD, with only a few scattered exceptions (Additional file 6). Specifically, significantly increased gene expression was detected in aging for CCR1 (HC, PCG), CXCL5 (HC), and CXCL16 (HC, PCG, SFG), while CXCL12 gene expression was decreased with aging in the HC (Table in Additional file 6: S6A). Only two chemokines showed a significant progressive pattern of change across aging and AD: CXCL16, which was upregulated in the EC and SFG, and CXCL14, which was progressively downregulated in the HC across aging and AD (Additional file 6: Table S6B). These data suggest that as a whole, chemokine genes are not extensively altered in aging or AD, even though microglia and macrophages appear to be activated, as reflected by upregulation of FcRs. This may be to the increased expression of S100A8 because it has been shown to cause a shift toward expression of activatory Fcgamma receptors on macrophages via toll-like receptor 4 [85].

Several mechanisms exist that function to curtail microglial activation. One important signaling system that appears to attenuate microglial proinflammatory responses is the fractalkine ligand and receptor pair (CX3CL1, CX3CR1 respectively). While in the periphery fractalkine signaling is associated with proinflammatory events, in the CNS, fractalkine is neuroprotective. In the CNS, fractalkine signaling mediates the communication between microglia and neurons, with the fractalkine receptor (CX3CR1) highly expressed on microglia and macrophages [86], and fractalkine constitutively expressed on neurons throughout the CNS [86]. While CX3CR1 expression was not altered in aging or AD, fractalkine gene expression was widely downregulated in aging in the EC, HC, PCG, and SFG (Additional file 6: Table S6A), with further downregulation in AD in the HC (Additional file 6: Table S6B). The decrease in fractalkine expression in the absence of parallel changes in the receptor likely represents a loss of communication between neurons and microglia/macrophages, and may promote an activated microglial/macrophage phenotype in the aging and AD brain.

Another system proposed to play a role in dampening the inflammatory response is CD163, a scavenger receptor expressed on microglia, monocytes and macrophages [87,88] with particularly high expression seen in macrophages of the ‘alternative activation’ phenotype [87]. In aging, CD163 was robustly elevated in the HC, PCG and SFG, with a trend toward continued upregulation in the SFG in AD (Additional file 5: Tables  S5A, B). CD163 can induce release of the anti-inflammatory interleukin IL-10 [89], which we found to be significantly elevated in the HC with aging and AD, as measured by qPCR (Figure 3A). Although the presence of ‘alternative activation’ phenotypes for macrophage and microglia in the AD brain has previously been observed [90], increased expression of CD163 in the AD brain is novel.

These data indicate that microglia and macrophages appear to have an activated phenotype in the aged brain with some additional activation progressing in AD, as reflected by upregulation of FcRs due to the activation of TLR4 by S100A8 [85]. Interestingly, chemokine genes as a whole did not show altered expression, likely reflecting that the state of inflammation present in the aged brain is of a low-grade nature, rather than a robust inflammatory state (such as would be present in cerebral infection). The decrease in fractalkine expression in the absence of parallel changes in the receptor likely represents a loss of communication between neurons and microglia/macrophages, and may promote an activated microglial/macrophage phenotype in the aging and AD brain. Finally, upregulation of CD163 and IL-10, which were particularly prominent in aging, likely provides some counterbalance to other gene changes that are proinflammatory.

### Aging- and AD-associated changes in expression for MHC class I and II genes

Another measure reflecting an activated innate immune response is the induction of MHC class I and II molecules. Although MHC I and II proteins are typically involved in presentation of foreign antigens processed from invading organisms, an increase in gene expression is also an early indication of activation of the innate immune response in the absence of foreign antigens, because MHC II genes are upregulated in sterile injuries or trauma. Further, MHC molecules are upregulated on microglia and macrophages in response to elevated levels of cytokines associated with inflammation, and in response to chronic pathology and neurodegeneration such as occurs in AD and amyotrophic lateral sclerosis (ALS) [91-94]. Indeed, increased gene expression of MHC II has been extensively documented in both AD and transgenic mouse models of AD [95].

Analysis of the classical MHC II and I genes revealed pronounced upregulation in aging and AD. Notably, aging was characterized by widespread upregulation of all MHC II subtypes (DP alpha, DP beta, DQ alpha, DQ beta, DR alpha, DR beta, HLA-DMA, HLA-DMB) across the four brain regions (Additional file 7: Table S7A), with continued upregulation of many of these genes in AD, particularly in the EC, PCG and SFG (Additional file 7: Table S7B). Of particular interest was the upregulation of HLA-DMA and HLA-DMB, two MHC class II genes that are critical for intracellular peptide loading of other MHC class II proteins on antigen-presenting cells. While in the HC, classical MHC class II genes showed an overall trend toward progressively increased upregulation in aging and AD, only one probe set met the statistical criteria of a ‘continuum’ gene. Finally, classical MHC class I genes (HLA-B, HLA-C) were upregulated with aging in the HC, PCG and SFG with progressively increased expression in the HC in AD, with no probe sets reaching significance in the EC in either aging or AD.

Interestingly, there was also a robust age-dependent increase in the nonclassical MHC I genes. HLA-E, HLA-F and HLA-G genes underwent pronounced upregulation in the aged brain, particularly in the HC, with HLA-E additionally upregulated in the PCG and SFG (Additional file 8: Table S8A). Gene expression for HLA-E and HLA-G showed progressive upregulation across aging and AD in limbic regions (both HC and EC), but not in the cortical regions (Additional file 8: Table S8B). Upregulation of HLA-E and HLA-G expression is emerging as a mechanism to protect target tissues from auto-aggressive inflammation, such as in conditions of chronic inflammation [96], and may function to provide inhibitory feedback to downregulate microglial activation.

Using the upregulation of MHC class genes as another readout of microglial activation, these data support the concept emerging from our data that microglia gain an activated phenotype that is initiated in the course of aging, and continues to progress in AD. It is likely that the chronic presence of low levels of proinflammatory cytokines accompanying innate immune activation is driving upregulation of MHC genes. Because the nonclassical MHCs are considered mediators of immune tolerance, the widespread upregulation of these genes in aging may be due to a chronic activation of the innate immune response.

### qPCR validation of immune gene expression changes in aging and AD

qPCR was undertaken in the HC from a subset of cases to validate the microarray data for several genes related to TLR signaling and regulation (specifically CD14, TLR2, TLR4, TLR7, MYD88, TOLLIP). In addition, qPCR was used to assess expression profiles of several genes for which gene expression was below the microarray sensitivity including the proinflammatory cytokines TNF-alpha, IL-Iβ, IL-6, the anti-inflammatory cytokine IL-10, and modulators of cytokine signaling including suppressor of cytokine signaling 3 (SOCS3) and interleukin-1 receptor-associated kinase 3 (IRAK3/IRAKM), a serine/threonine kinase that helps mediate signaling from TLRs and IL-1 receptor family members. Finally, for assessment of inflammasome-related gene expression, qPCR was undertaken in the SFG for NRLP3 and ASC. A subset of cases used in the microarray was used for qPCR analysis based on tissue availability.

**Figure 4:**
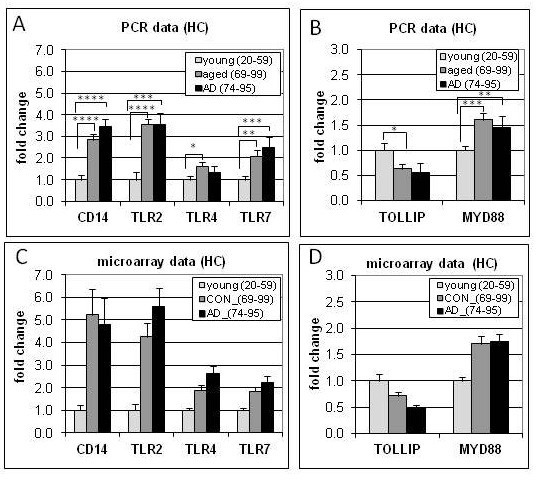
**Gene expression profiles in young, aged, and Alzheimer’s disease (AD) samples using hippocampal tissue show similar patterns of change with qPCR and microarray analysis.** qPCR (**A**,**B**) demonstrated significant gene upregulation of CD14, TLR2, TL4, TLR7, MYD88 and downregulation of TOLLIP in aging, confirming microarray results (**C**,**D**). **** *P* < 0.0001, ***P < 0.005, **P < 0.01, *P < 0.05.

The expression profiles across aging and AD obtained by qPCR for CD14, TLR2, TLR4, TLR7, MYD88, and TOLLIP were in good agreement with the expression profiles determined using microarrays (Figure 4). For comparison purposes, microarray data is shown for the same set of cases as were used for qPCR. Analysis of cytokine expression profiles using qPCR revealed increased expression with aging for IL-Iβ, IL-6, TNF-alpha, and the anti-inflammatory cytokine IL-10. Intriguingly, IL-10 showed a modest increase with aging and a more pronounced increase following the transition to AD (Figure 3A). In addition, analysis of gene expression profiles for modulators of cytokine signaling revealed robust gene upregulation of IRAK3 and SOCS3 with age, but no further increase with AD (Figure 3). Finally, no significant gene expression changes were detected for the inflammasome-related genes NRLP3 and ASC, either in aging or AD (Figure 3B). Overall, the patterns of gene expression change detected using qPCR paralleled the general pattern that emerged from the microarray analysis of immune/inflammation genes as a whole, with the greatest gene expression changes generally occurring with aging rather than in the transition to AD.

## Discussion

While there is extensive data on immune gene responses in the AD brain [2], less is known about the brain responses of these genes across the lifespan in normal aging, particularly for the innate immune system. There is an increasing appreciation that innate immunity can be activated in the CNS in response to cellular stress or injury without activation of the adaptive arm of the immune system [54,97-102], and importantly, that the innate immune response can be activated by a number of endogenous factors that may accumulate in the brain with aging and AD. Using a microarray approach, we have comprehensively investigated immune gene expression in four brain regions in cognitively normal aging across the adult lifespan (20 to 99) and in AD, with a particular focus on subclasses of the innate immune response.

Several novel concepts emerge from this analysis. First, our data reveal that immune/inflammation-related genes show major changes in gene expression over the course of cognitively normal aging, and that the extent of gene response is far greater in aging than in AD. Of the 759 immune-related probesets interrogated on the microarray, approximately 40% are significantly altered (*P* < 0.01) in the SFG, PCG and HC with increasing age, with the majority upregulated (64 to 86%). In contrast to the extensive gene response in aging, far fewer immune/inflammation genes were significantly changed in the transition to AD (approximately 6% of immune-related probe sets), with gene responses primarily restricted to the SFG and HC. Second, relatively few significant changes in immune/inflammation genes were detected in the EC either in aging or AD, although many genes in the EC showed similar trends in responses as in the other brain regions. Third, a subset of immune/inflammation genes undergo a progressive pattern of change across aging and AD, with these genes predominantly showing progressive upregulation. Interestingly, very few genes in the PCG underwent a progressive pattern of change across aging and AD (some exceptions included complement C5AR1 and several MHC class II genes), even though extensive change occurred in this brain region during aging. The PCG thus appears to be refractory to change during the transition to AD, which may be related to the relative sparing in this brain region from AD-related neuropathology. Finally, our data reveal that immune/inflammation genes undergo gender-specific patterns of response in aging and AD, with the most pronounced differences emerging in aging. In particular, the HC shows major changes in normal aging that are most prominent in females, while the SFG shows greater age-related changes in males. These results support accumulating evidence of a gender effect on brain aging, which have recently been documented at the gene expression level [28], morphometric level [103,104] and level of cortical connectivity networks [105].

Strikingly, functional analysis of the immune genes significantly changed with aging or AD revealed that many of these genes were associated with the innate immune response. Key components of the innate immune system are complement, TLRs, inflammasomes, and scavenger and immunoglobulin receptors [61]. Our data demonstrate that in aging, there are major changes across nearly all these gene classes in the HC, PCG, and SFG, with gene responses overwhelmingly favoring increased activation. A subset of these genes showed progressively more change with the transition to AD, particularly in the HC and SFG. Because activation of the innate immune response induces release of proinflammatory cytokines and key co-stimulatory molecules, these gene changes indicate a broad-based increase in a proinflammatory environment that is initiated with normal aging and that continues to increase to a lesser degree following the transition to AD.

A concept that has gained traction in recent years is that changes that occur during normal aging may prime the CNS for subsequent development of neurodegenerative disorders [29-31]. Conditions in the aged brain bear similarity to a chronic injury environment, including accumulation of a variety of endogenous factors (fibrillar Aβ, calprotecin, and proinflammatory cytokines) that can activate various arms of the innate immune system and trigger a feed-forward mechanism driving chronic innate immune activation. Inefficient clearance of aberrant proteins, a process that becomes increasingly defective with age [106] may represent an initiating factor upregulating innate immune activity in the brain. In addition, accumulation of Aβ acts as a low-grade irritant that can trigger the innate immune system via several mechanisms, including complement activation [37], activation of TLR signaling [39-48], and activation of the inflammasome [49]. Moreover, impaired clearance of Aβ may be related to deficits in beclin 1, an autophagy-related protein that has been shown to decrease Aβ accumulation in mice [107-109]. In our dataset, beclin 1 gene expression was decreased in aging in the HC, PCG, and SFG with continued decline in the SFG in AD, consistent with recent findings that beclin-1 protein in the mid-frontal cortex is reduced in early AD [107-109]. We speculate that the beclin 1 deficit that develops during normal aging may contribute to Aβ accumulation, which can in turn activate complement, TLRs, and the inflammasome (when in a fibrillar form), with consequent release of potent inflammatory peptides and proinflammatory cytokines [48,49,110]. Aβ is likely to be both an initiating trigger and chronic driver of innate immune activation [111], consistent with growing data suggesting that MCI cases with high Aβ are at increased probability for converting to AD [112].

Our data showing widespread caspase-1 upregulation with aging suggest that the inflammasome may be active in the aged brain. The increased expression of calprotectin, which along with Aβ can trigger TLR activation, may establish a feed-forward mechanism for continued activation of the innate immune system and perpetuation of a chronic proinflammatory environment in the aged brain. Further, inflammasome activation of caspase-1 has been reported to initiate unconventional protein secretion (for example, independent of the endoplasmic reticulum-Golgi secretory pathway) to export cytokines from the cell, including IL-1β and IL-18, allowing these proinflammatory molecules to engage their extracellular receptors [113,114]. Caspase-1-induced unconventional protein secretion may facilitate the cellular efflux of other cytoplasmic proteins, including misfolded tau and alpha-synuclein, thereby participating in the propagation of these proteinopathies [115,116], which in turn further contribute to innate immune activation.

At the same time, a number of counteractive measures to attenuate a proinflammatory environment exist. Our microarray data reveal that several of these protective mechanisms appear to be engaged in the brain in aging and AD, including upregulation of CFH, CFHR1 and clusterin to curtail complement activation, upregulation of the anti-inflammatory cytokine IL-10, upregulation of CD163, and upregulation of nonclassical MHCI genes (HLA-E and HLA-G). However, our microarray data also revealed that some of the measures to attenuate a proinflammatory environment are downregulated with aging and AD, for example TOLLIP, which attenuates TLR-signaling, and fractalkine in neurons, which curtails microglial activation. TOLLIP underwent progressively pronounced downregulation in aging and AD, notably in brain regions that are vulnerable to decline in aging and AD. In parallel, fractalkine gene expression was widely downregulated in all brain regions examined, with additional downregulation in AD in the HC. Several lines of evidence support an important neuroprotective role for fractalkine signaling, including the demonstration in three different *in vivo* models that deficiency of the fractalkine receptor (CX3CR1) alters microglial responses and results in significant neurotoxicity [117], and impairs hippocampal cognitive function and synaptic plasticity [118]. The decline in fractalkine gene expression potentially contributes to decreased neuronal control of microglial activation, and in parallel, downregulation of TOLLIP suggests that the brakes on TLR signaling are less accessible with age and AD, both of which would contribute to driving a chronic proinflammatory state. These data suggest that one therapeutic approach to interrupt or attenuate the cycle of chronic innate immune activation may be to develop interventions that counteract downregulation of these protective mechanisms.

Finally, our data reveal an aspect of microglia activation present in aging that may have relevance to the adverse cerebrovascular events reported with the anti-Aβ immunotherapy clinical trials [119]. Namely, the aging brain shows widespread increased expression of activating FcRs (FcRI, IIa, IIIb). Because regulation of antibody-mediated immune responses is crucial to prevent uncontrolled inflammation and tissue damage, both activating and inhibitory FcRs are generally expressed by cells. However, our data revealed that in the aged and AD brain, gene expression for activating FcRs was upregulated in the absence of a parallel response of inhibitory FcRs. One factor that may contribute to the specific upregulation of activating FcRs may be related to the age-related increased gene expression of calprotectin, which can shift FcR expression toward activating Fcgamma receptors on macrophages via toll-like receptor 4 [85]. The upregulation of activating FcRs may be detrimental when antibodies or immune complexes are present in the brain, especially when these responses are not appropriately regulated by inhibitory FcRs. This may be particularly relevant when active or passive anti-Aβ immunotherapy is administered to elderly individuals who have substantial amyloid burden in the brain. Aβ-antibody immune complexes can initiate microglia and perivascular macrophage activation via FcRs, as well as trigger complement activation and release of inflammatory mediators, thereby potentially contributing to the adverse cerebral vascular events that have plagued the immunotherapy clinical trials [119,120].

Our data are consistent with previous immunohistochemical studies of AD brain tissue, which have shown many of the classic features of immune/injury-mediated cellular damage including increases in proinflammatory cytokines, expression of MHC class I and class II antigens on microglia, and evidence of complement activation within thioflavin-positive neuritic plaques [2,55,56,121,122]. Importantly however, our data emphasize the fact that the majority of these changes in immune/inflammation-related genes in the brain occur long before the earliest clinical signs of cognitive decline, with only modest additional change occurring in AD. These findings help clarify recent epidemiologic evidence that midlife long-term use of NSAIDs delays onset of AD, and findings from a randomized clinical trial (Alzheimer's Disease Anti-inflammatory Prevention Trial (ADAPT)) that NSAID treatment of asymptomatic individuals reduces AD incidence with long-term use (two to three plus years) while NSAIDs provide no benefit in patients with symptomatic AD and have an adverse effect in later stages of AD [123]. These results are consistent with our data demonstrating that much of the immune activation has been chronically present long before clinical symptoms of AD become apparent. Overall, these findings provide important basic knowledge on the state of immune activation in the brain with aging and AD, data that will be particularly useful for tailoring therapeutic approaches that target inflammation to slow cognitive decline in aging and AD.

## Conclusions

Our data reveal that the aging brain is characterized by widespread upregulation of genes reflecting activation of microglia and perivascular macrophages, with the upregulation of essentially all pathways of the innate immune system coupled with a downregulation of select factors (TOLLIP, fractalkine) that when present curtail microglial/macrophage activation. Activation of the innate immune response is initially likely to be beneficial. However, long-term innate immune activation causes chronic proinflammatory conditions and release of endogenous factors (Aβ, calprotectin, proinflammatory cytokines) that can drive destructive cascades. Unexpectedly, the extent of innate immune gene upregulation in AD was modest relative to the robust response apparent in the aged brain, consistent with the emerging idea of a critical involvement of inflammation in the earliest stages, perhaps even in the preclinical stage, of AD.

We hypothesize that with aging, the brain accumulates levels of multiple endogenous and exogenous factors that act as low-grade irritants continuously reinforcing microglia activation and priming microglia responses. AD ensues when the net effect of these factors surpasses a certain threshold, partnered with reduced capacity to temper microglial reactivity. Aβ, in conjunction with increased levels of harmful endogenous factors, impaired growth factor signaling by proinflammatory cytokines [124], metabolic deficits [125], and other factors, ultimately converge to exceed the threshold for the onset of AD. Ultimately, our data suggest that an important strategy to maintain cognitive health and resilience involves reducing chronic innate immune activation, but that this intervention should be initiated relatively early in aging (by approximately 50 years of age), and prior to clinical signs of cognitive difficulty.

## Competing interests

The authors declare that they have no competing interests.

## Authors’ contributions

PDC, JR, and CWC conceived the research designed; NCB and VP performed the research; NCB and CWC analyzed data; and NCB, DHC, AJT, and CWC wrote the paper. All authors have read and approved the final version of the manuscript.

## Supplementary Material

Additional file 1 **Table S1.** Case information and brain regions for young, aged and AD cases. Click here for file

Additional file 2 **Table S2.** Relative expression values for complement signaling genes and associated probe sets. Click here for file

Additional file 3 **Table S3.** Relative expression values for toll-like receptor signaling genes and associated probe sets. Click here for file

Additional file 4 **Table S4.** Relative expression values for inflammasome-related genes and associated probe sets. Click here for file

Additional file 5 **Table S5.** Relative expression values for genes and probe sets related to scavenger and Fc function. Click here for file

Additional file 6 **Table S6.** Relative expression values for chemokine genes and associated probe sets. Click here for file

Additional file 7 **Table S7. **Relative expression values for MHC II signaling genes and associated probe sets. Click here for file

Additional file 8 **Table S8.** Relative expression values for MHC I genes and associated probe sets. Click here for file
